# High-Energy Ball Milling for High Productivity of Nanobiochar from Oil Palm Biomass

**DOI:** 10.3390/nano12183251

**Published:** 2022-09-19

**Authors:** Lawrence Yee Foong Ng, Hidayah Ariffin, Tengku Arisyah Tengku Yasim-Anuar, Mohammed Abdillah Ahmad Farid, Mohd Ali Hassan

**Affiliations:** 1 Laboratory of Biopolymer and Derivatives, Institute of Tropical Forestry and Forest Products, Universiti Putra Malaysia, Serdang 43400 UPM, Malaysia; 2 Department of Bioprocess Technology, Faculty of Biotechnology and Biomolecular Sciences, Universiti Putra Malaysia, Serdang 43400 UPM, Malaysia; 3 Nextgreen Pulp & Paper Sdn. Bhd., Green Technology Park, Paloh Inai, Pekan 26600, Malaysia; 4 Department of Biological Functions Engineering, Gradute School of Life Science and Systems Engineering, Kyushu Institute of Technology, Fukuoka 808-0196, Japan

**Keywords:** nanobiochar, biocarbon, high-energy ball milling, biomass utilization, organic nanofiller

## Abstract

The current production method of nanobiochar (NBC), an emerging, environmentally friendly nanocarbon material, is tedious and lengthy. Therefore, in this study we aimed to improve the productivity of NBC via high-energy ball milling by manipulating the grinding media and processing time. The particle size distribution of the resulting NBC measured using dynamic light scattering showed that grinding media with steel balls of different sizes were more effective at producing NBC than small uniform steel balls, which failed to produce NBC even after 90 min of milling. Average NBC particles of around 95 nm were achieved after only 30 min of ball milling, and the size was further reduced to about 30 nm when the milling was prolonged to 150 min. Further prolonging the milling duration led to agglomeration, which increased the size of the biochar nanoparticles. The thermogravimetric analysis (TGA) data showed that the duration of milling and particle size did not cause noticeable differences in the thermal stability of the NBC. Based on the FTIR analysis, the chemical structure of the NBC was not affected by the ball milling. The results showed that 60 min of high-energy ball milling is sufficient to produce NBC particles of 75 nm, with a large surface area and high thermal stability. This could prove beneficial in a myriad of applications, ranging from agriculture to composite fabrication.

## 1. Introduction

Malaysia is one of the world’s largest producers of palm oil, making up about 28% of the world’s palm oil production and 9.5% of the world’s total oil and fat production [1]. This contributes to Malaysia’s significant agricultural waste production rate. In Malaysia, an estimated 12 Mt of agricultural biomass are produced each year, with oil palm biomass making up 77% of the total lignocellulosic waste that can potentially be utilised for value-added products [2]. Oil palm empty fruit bunches (OPEFB) make up a significant portion of the waste generated from the oil palm industry. It is estimated that for every 1 ton of palm oil produced, approximately 1 ton of OPEFB will be generated as a by-product [3]. Therefore, much effort has been put into utilising OPEFB by using them as a fuel for energy production or by converting it into other forms of raw materials such as biochar [4,5,6].

Biochar is a sustainable material produced from the carbonisation of biomass, primarily from agricultural waste [7]. Agricultural by-products such as OPEFB, which make up a significant portion of the waste generated by the palm oil industry, have been proven to be suitable for the production of biochar via pyrolysis [8]. Due to the removal of volatile compounds during pyrolysis, biochar has a porous structure [9]. This porous structure allows biochar to be commonly used for environmental and agricultural purposes, where the carbonaceous material is able to adsorb contaminants or heavy metals and retain fertilisers for the controlled release of nutrients into the soil [10,11]. A lesser known property of biochar is its high thermal stability due to the nature of pyrolysis [12]. The high thermal stability of biochar allows it to be used as a filler in polymers to produce heat resistant composites, further broadening the range of applications for biochar. The addition of biochar into polymers has been an emerging trend, with the intention of improving the thermal stability of these materials. A study by Das et al. [13] showed an increase in the thermal stability of polypropylene when biochar was added, with a delay in the maximum decomposition temperature. 

Smaller particle sizes have long been known to increase the surface-area-to-volume ratio of a material. In the case of biochar, this increase in the surface-area-to-volume ratio has been proven to be beneficial in the field of agriculture, since it often results in advantages such as better water retention capability in soil, higher nutrient uptake, and overall better plant performance [14,15]. Similarly, for reinforcing materials, a larger surface area would lead to better interaction between the filler and the polymer matrix. This has been observed in many cases involving both particulate and fibrous additives, where the same additive at the nanoscale led to improved properties in the composite. Carbonaceous materials such as biochar are no exception, as described in a study by Richard et al. [16]. In this study, it was found that biochar with smaller particle sizes (as small as 45 nm) led to a significant improvement in the properties of PVA in terms of the mechanical properties and dielectric constants. It was shown in the report that the void fraction was lower for biochar with smaller particle sizes, and the dispersion of the sub-micron biochar particles was more uniform. This could also be related to the surface characteristics of NBC, which has more polar groups exposed than the larger-particle biochar, meaning it is more compatible with hydrophilic PVA [17].

While NBC particles are preferred over the larger version of biochar in many cases, the limitation of utilising NBC lies in its production. Currently, the process of converting biochar into NBC is tedious. The current NBC production methods either involve milling or pre-treatments that require long durations. In one study, biochar produced from rice husk was converted into NBC by means of planetary ball milling, and while the authors were successful in producing NBC, the milling process took a duration of 30 h to reduce particle sizes below 100 nm [16]. Another study by Naghdi et al. [18] also used planetary ball milling to convert biochar into NBC. This time, they managed to reduce the milling time to 100 min, but the method required pre-conditioning of the biochar at −80 °C for 24 h. Li et al. [19] described the extraction of NBC from biochar samples via planetary ball milling for 2 h followed by repeated sonication and centrifugation, resulting in two layers of biochar, with the upper layer consisting of smaller micro- or nanobiochar particles.

It is noticeable that while planetary ball milling is a viable method for breaking larger biochar pieces down to the nanoscale, the process is time-consuming, often requiring many hours of milling or pre-conditioning. An alternative ball milling technique would be useful in high-energy ball mills. When compared to planetary ball mill, which have a mechanism where the vials and the disk move in counter-rotational directions parallel to the ground, high-energy ball mills move in a more complicated ‘figure of eight pattern consisting of both horizontal and vertical movements of the grinding media [20]. This complex range of motion, as well as the high frequency of the system, allows the grinding media in the mill to generate tremendous impact force on the sample material when compared to planetary ball milling, which is more commonly used in the production of NBC [21]. However, there are limited studies on the production of NBC using a high-energy ball mill. Another possible factor in extended milling durations may be the raw material itself. The information on the mechanical properties of biochars produced from different raw materials is scarce. However, there are studies that show that biochar produced from woody materials such as soft wood from spruce or fir trees would be denser and harder and would require more force to be broken down as compared to the less dense structure of biochar produced from a non-wood material such as hemp [22]. Since OPEFB are also a type of non-wood material, it is possible that the resulting OPEFB biochar would be easier to break down as compared to woody biochar.

Since NBC is an emerging nanomaterial with various potential applications, this study was conducted to investigate the effects of the process parameters on the production of OPEFB-NBC using high-energy ball milling instead of conventional planetary ball milling, with the aim of producing NBC from biochar without the need for pre-conditioning or pre-treatment steps in a shorter duration.

## 2. Materials and Methods

### 2.1. Materials

The biochar was produced from the pyrolysis of OPEFB sourced from a local palm oil mill (Seri Bandar Palm Oil Mill, Malaysia) at a temperature of 500 °C for 25 min in a tubular furnace (Dentsply Ceramco, York, PA, USA) with a nitrogen flow rate of 258 mL/min; this is the modified method of biochar production used by Lawal et al. [23]. The 95% ethanol was purchased from Systerm Chemicals, Shah Alam, Malaysia.

### 2.2. Sample Preparation and Ball Milling

The biochar was subjected to mechanical grinding with a commercial food blender to produce the biochar powder. The biochar powder was then ground into NBC in a ball mill using a SPEX 8000D (Metuchen, NJ, USA) high-energy ball mill with two 65 mL vials. This process was conducted at ambient temperature. Mixed sizes (2.5–11 mm in diameter) of hardened steel balls (grinding media) were used. In each vial, 50 g of grinding media and 5 g of biochar powder were weighed to give a ball-to-powder-mass ratio (BPR) of 10:1. To compare the effect of the size of the grinding media on the particle size of the biochar, uniform hardened steel balls measuring 2.5 mm and 11 mm in diameter were also used in separate runs with the same BPR. The biochar was milled for various milling durations (Table 1) with a fixed milling speed of 1725 rpm. The ball milling was performed at maximum intervals of 90 min, with a resting time of 30 min between each interval.

### 2.3. Morphology

Field emission scanning electron microscopy (FESEM, Sirion 200, FEI, Eindhoven, The Netherlands) was used to observe the morphology of the biochar particles. The samples were sputter-coated with gold prior to observation. The transmission electron microscopy was performed using a JEOL JEM 2100F Field Emission TEM (JEOL, Tokyo, Japan). The samples were first dispersed in ethanol to a concentration of 0.025 wt% and then ultrasonicated. A small droplet was placed on a copper grid and left to dry at room temperature.

### 2.4. Particle Size Distribution

The particle size distribution of the biochar samples was analysed via dynamic light scattering (DLS) using a Zetasizer NanoS instrument (Malvern Instruments, Malvern, UK). The samples were prepared for DLS by placing 1 mg of biochar into 200 mL of distilled water with 1% ethanol [18]. The samples were then stirred using a magnetic stirrer for 20 min before sonication in an ice water bath for an additional 20 min.

### 2.5. Thermal Stability

A thermal stability analysis of the biochar samples was performed using a thermogravimetric analyser (TGA) (TGA 4000, Perkin Elmer, Waltham, MA, USA). Approximately 8.5 mg of each biochar sample was loaded onto a ceramic pan. The samples were held at 50 °C for 1 min before heating up to 700 °C with a heating rate of 10 °C/min. A nitrogen gas flowrate of 10 mL/min was applied. The TG and derivative thermogravimetry (DTG) curves were obtained from each run.

### 2.6. Crystalline Properties

The crystal profiles of the ball-milled biochar samples were obtained using an X-ray diffractometer (XRD) (Rigaku Miniflex II, Tokyo, Japan) equipped with a radiation source of Cu-Kα, λ = 0.15406 nm. The analysis was done with voltage of 50 kV, a current of 1 mA, and 2θ = 3°–80°. The full-width at half-maximum (FWHM) of each peak was obtained using Origin, version 2019b; Software For Data Analysis And Graphing; OriginLab: Northampton, MA, USA, 2019 and the D-spacing was calculated in accordance with Bragg’s law, as shown in Equation (1):

nλ = 2dsinθ(1)

where n represents the order of reflection and λ is the wavelength of the X-ray from the Cu source equating to 0.15406 nm, while d indicates the D-spacing or lattice spacing and θ is the peak position acquired from the XRD profiles.

Additionally, the Scherrer equation was used to determine the crystallite dimensions such as the crystallite diameter, L_a_, and crystallite stacking height, L_c_, as shown in Equation (2):

D = (Kλ)/((β × cosθ))(2)

where D is the crystallite diameter (L_a_) or crystallite stacking height (L_c_) and K is the Scherrer constant corresponding to 0.94 or 1.84, depending on the calculations of the crystallite diameter or crystallite height, respectively. Here, β refers to the full-width at half-maximum (FWHM) of the biochar peaks, which can be calculated from the XRD profiles; λ and θ represent the wavelengths of X-ray from Cu source and peak position, respectively, as described in Equation (1).

### 2.7. Fourier Tranform Infrared Spectroscopy (FTIR)

An FTIR analysis was performed using a Nicolet iS10 ATR-FTIR system (Thermo Fisher Scientific, Waltham, MA, USA). The analysis was performed in the range of 4000–500 cm^−1^ with a resolution of 4 cm^−1^ and 16 scans per sample. All samples were dried in an oven at 60 °C overnight prior to analysis to remove the interference from the hydrogen bond formation of the samples with water.

## 3. Results and Discussion

### 3.1. Morphology

**Figure 1:**
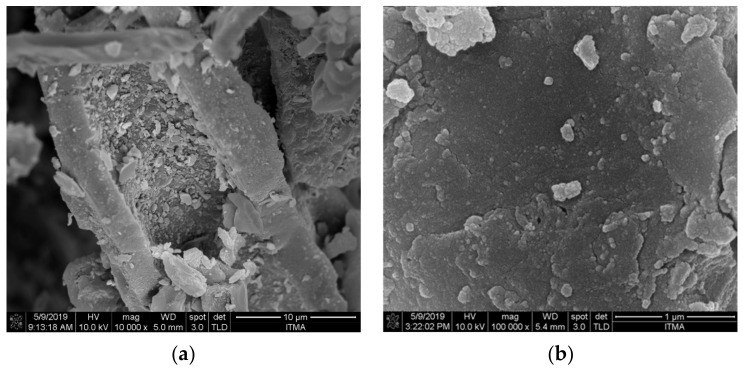

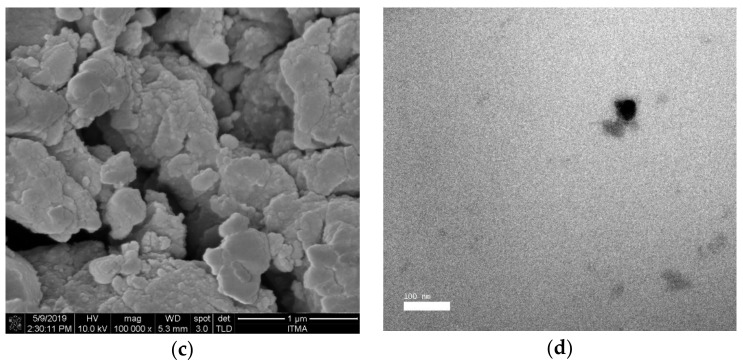
FESEM micrographs of biochar samples before and after ball milling; (**a**) OPEFB biochar (10k × magnification); (**b**) 60NBC (100k × magnification); (**c**) 360NBC (100k × magnification); (**d**) TEM micrograph of 60NBC (25k × magnification).

Figure 1 depicts FESEM micrographs of the OPEFB biochar before and after ball milling for various durations. The OPEFB biochar refers to the biochar collected after the pyrolysis of OPEFB before the mechanical grinding and ball milling were performed. From Figure 1a, it can be seen that the OPEFB biochar has a rod-like morphology representative of the physical structure of the OPEFB fibres. After ball milling, the FESEM images show an apparent decrease in the size of the biochar particles. This is especially true for 60NBC, for which a large portion of the particles were within the 100 nm size range requirement for nanomaterials existing as aggregates. To better observe the individual particles and to verify the dimensions of the NBC, TEM was also conducted on 60NBC. The TEM image of 60NBC shown in Figure 1d reveals the existence of particles well below 100nm. It would seem that the morphology of the NBC particles has been transformed into rough polygonal shapes rather than the long rod-like particles of the original OPEFB biochar. A similar NBC morphology was also observed in another study involving planetary ball milling [24]. Conversely, 360NBC shows a significant amount of agglomeration of the biochar particles that is typical of prolonged ball milling. This is due to the repeated trapping and compaction of the NBC particles in between the grinding media [25]. Biochar particles are also known to contain functional groups that are capable of forming hydrogen bonds with each other, which could be exacerbated by longer durations of ball milling [26]. According to a study by Bakhtiar et al. [27], OPEFB biochar pyrolysed at temperatures between 400 °C and 600 °C gave rise to more OH functional groups as opposed to higher temperatures of 800 °C and above. When broken up into smaller particles, the higher total surface area of the particles could increase the tendency of the hydrogen bonds to form between the surfaces of these particles and could lead to the agglomeration of these particles.

### 3.2. Particle Size Distribution

Since biochar conforms into rough polygonal-shaped particles as an effect of ball milling, as shown in the FESEM images (Figure 1), the hydrodynamic diameter of the biochar particles was used to estimate the particle size distribution using dynamic light scattering (DLS). DLS measures the intensity fluctuations caused by the Brownian motion of different sized particles as they move randomly through a medium.

**Figure 2:**
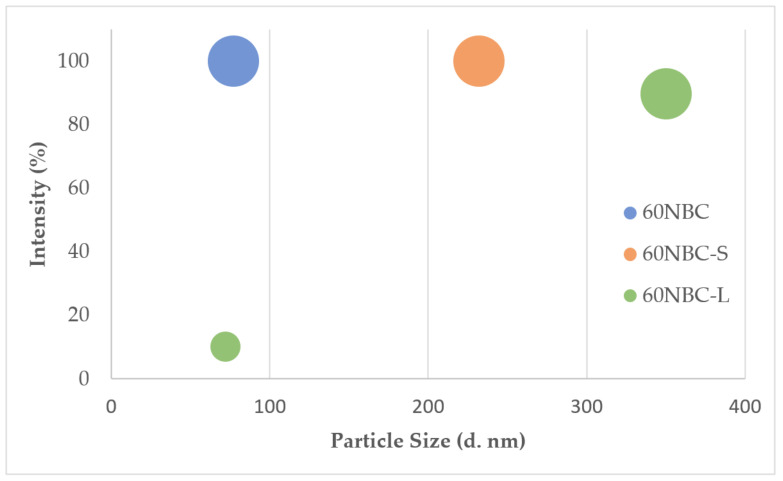
The effect of the grinding media on the particle size distribution of the NBC.

Figure 2 shows the effects of the grinding media and milling duration on the particle size distribution of the NBC. It is apparent that the grinding media with a mix of different sized steel balls (60NBC) were more effective in transforming the BC into small particle sizes compared to the uniformly sized small steel balls and large steel balls (60NBC-S and 60NBC-L). Here, 60NBC-S did not convert the BC into NBC, while only a small portion of the 60NBC-L particles were below 100 nm. As seen from the particle size distribution in Table 2, the average particle size for 60NBC-S was approximately 232 nm, while most of the 60NBC-L particles were 350 nm in size. This observation can be explained via the theory of linear momentum and impulse [28]. There are several factors contributing to the changes in particles during collision, and these include the mass, velocity, and concentration of the reactant; the number of collisions; and the surface area. When the mass ratio of the steel balls to the particles is fixed, it can be easily understood that there will be more small steel balls needed to achieve the mass ratio compared to the process which uses larger steel balls. Even though larger steel balls will be able to create higher forces due to their mass, in the case of collisions to produce nano-sized materials, the high concentration of the small steel balls is important, as it will contribute to the higher number of collisions and higher surface area exposed for the collisions to occur. Hence, 60NBC-S was able to produce a smaller NBC compared to 60NBC-L. On the other hand, the use of mixed small and large steel balls in a process will contribute to a superior effect during NBC production, since the process would have the benefits from both the small and large steel balls. This can be seen from our results for 60NBC, which show that the average size distribution was approximately 77 nm.

**Figure 3:**
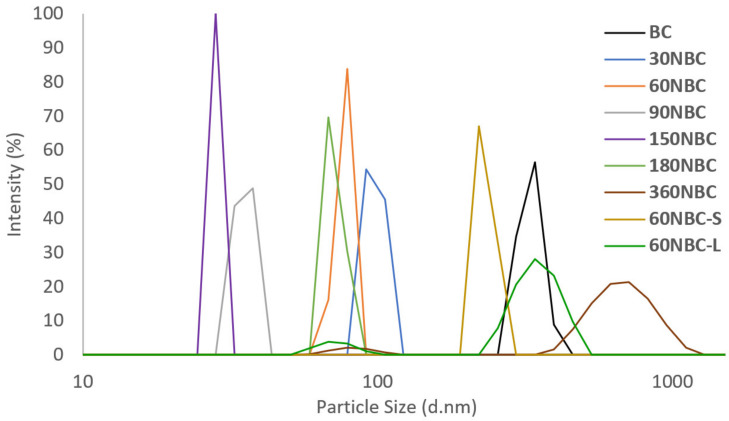
The particle size distribution of the biochar and NBC based on intensity.

Our detailed analysis on the effect of the milling duration on the particle size distribution is summarized in Figure 3 and Table 2. The unmilled biochar after mechanical grinding (BC) had an initial diameter of approximately 331 nm. After 30 min of ball milling, the diameter of the biochar decreased to an average diameter range of 90 to 100 nm. The NBC continued to decrease in size as the duration of the ball milling increased up to 180 min, where there was a noticeable increase in the size of the particles. This was due to the agglomeration of the biochar particles, as mentioned before. With the longer milling duration of 360 min, the NBC particles fluctuated in size, revealing a large portion of the particles with an average particle size of 350nm. This was possibly due to an increased occurrence of agglomeration, as shown in the FESEM image (Figure 1c).

### 3.3. Thermal Stability

**Figure 4:**
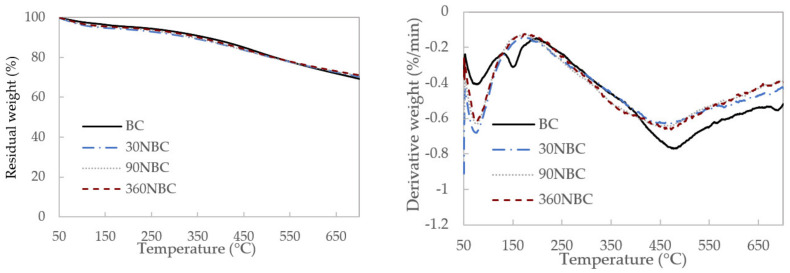
(**a**) The TGA and (**b**) DTG profiles of biochar (BC) and NBC samples.

The TGA and DTG curves of each individual sample are displayed in Figure 4. All samples started with an initial drop in mass within the first 100 °C. This could be attributed to the loss of water that was absorbed by the biochar due to its high absorption capability. All biochar and NBC samples were thermally stable, with more than 70% weight residue even at 700 °C. The DTG curves in Figure 4b for all samples also follow a similar pattern, and the reduction in particle size showed minimal effect on the rate of mass loss. This was because the cellulose, hemicellulose, and the majority of the lignin in the OPEFB had already been carbonised during the pyrolysis process, leaving behind thermally stable biochar with a high carbon content. According to previous studies on the production of biochar via pyrolysis, cellulose and hemicellulose are degraded at around 200–300 °C, while lignin continues to degrade at temperatures above 600 °C [29,30]. This could explain the decrease in weight depicted in Figure 4 as the temperature gradually increases.

### 3.4. Crystallinity

**Figure 5:**
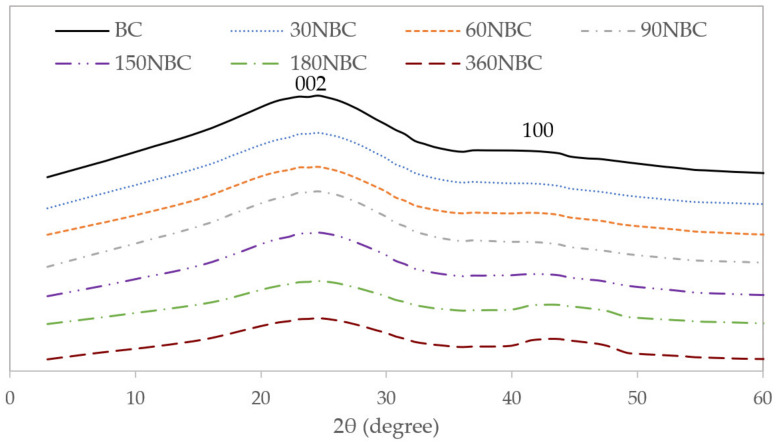
The XRD profiles of BC and NBC samples at different milling durations.

Figure 5 shows the XRD profiles of the biochar, as well as the various NBC samples. The two broad diffraction peaks are representative of the 002 and 100 plane reflections, which are characteristic of turbostratic biochar. Turbostratic biochar is a type of carbon material, where unlike graphitic carbon, which has neatly stacked graphite layers, turbostratic biochar contains bent, unstacked graphite layers [31]. While it does not contain large crystallites like in graphitic materials, turbostratic biochar still contains small regions of disordered graphitic layers, for which the dimensions can be calculated from the XRD profiles [32].

According to the overall crystal dimensions of the respective samples in Table 3, it seems that the ball milling of the biochar had some effect on the overall dimensions of the biochar crystal structure. It is evident that an increase in milling durations slightly decreases the average crystal dimensions, which is expected of high energy ball milling due to the high degree of mechanical forces used to break down the larger biochar particles [33,34]. This is proven by the decrease of approximately 0.2 nm in L_a_ after the initial 30 min of ball milling. Prolonging the duration of the ball milling beyond 30 min continued to decrease the La but to a lesser extent. In contrast, the duration of the ball milling had no significant effect on the L_c_ of each sample. However, there was a slight shift in the 2θ angles, especially for the 100 plane, shifting from 40.53° for BC to 43.88° for 360NBC. The shift in peak position, together with the broadening of the 100 peak and the decreasing D-spacing (002), is due to the residual stress to the biochar turbostratic layers caused by the ball milling [35].

### 3.5. FTIR Analysis

**Figure 6:**
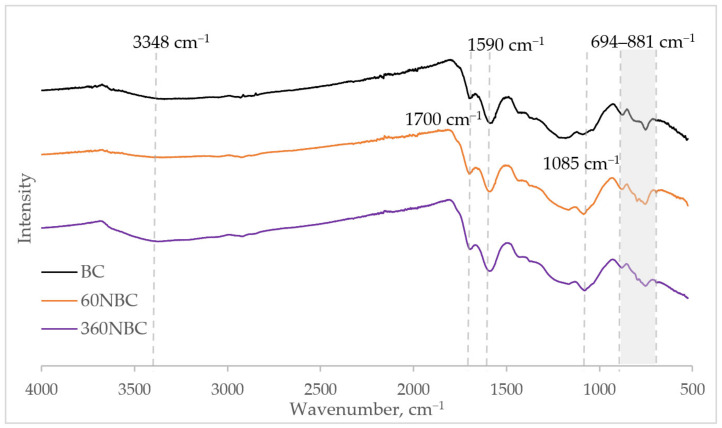
FTIR spectra of BC, 60NBC, and 360NBC.

The functional groups of BC, 60NBC, and 360NBC were analysed via FTIR spectroscopy to determine whether the ball milling chemically affected the biochar particles. The FTIR spectra of the aforementioned samples can be found in Figure 6. All three samples showed near identical FTIR spectra, with bands characteristic of biochar. The distinct band at 1700 cm^−1^ corresponds to the carboxylic C=O stretching vibrations. The band at 1590 cm^−1^ is indicative of C=C aromatic and alkene stretching vibrations. The band at 1085 cm^−1^ is attributed to the phenolic C-O stretching. The bands in the region between 694 and 881 cm^−1^ are caused by aromatic C-H stretching. There is also a wide band around 3348 cm^−1^ that belongs to the O-H stretching of the carboxyl groups. It is noticeable that the band for the O-H stretching was slightly more prominent in 360NBC. Since all of the samples were dried in a drying oven prior to analysis, it could be that the increase in intensity of the O-H band was due to the formation of hydrogen bonds between the NBC particles, which caused the agglomeration to occur. The lack of new bands or shifts in bands and the largely similar patterns of the FTIR spectra for BC, 60NBC, and 360NBC indicate that the ball milling does not cause chemical changes to the structure of the biochar and remains a mainly mechanical method of NBC fabrication.

## 4. Conclusions

Biochar was successfully produced from OPEFB and converted into NBC via high-energy ball milling at 30 min. This short duration of ball milling managed to produce NBC particles of 98 nm, with even smaller NBC particles produced after prolonged ball milling durations of up to 150 min. The NBC maintained its thermal stability, having more than 70% residual weight at 700 °C after ball milling. The crystallite dimension of the NBC was affected slightly, with an initial decrease in L_a_ after the ball milling of the raw biochar. However, after the initial decrease in L_a_, there was no significant change in the crystallite dimensions with prolonged milling durations. The FTIR analysis also showed that high-energy ball milling does not affect the chemical structure of biochar. Based on the data compiled in this study, high-energy ball milling is capable of producing NBC particles in short durations of 30–150 min. As a carbon-based nanomaterial, the NBC produced in this manner has high potential to be used in polymer bionanocomposites, and is expected to improve the mechanical, thermal, and crystallization properties of the polymer bionanocomposites. We are currently evaluating the NBC for the mentioned applications, and the results are expected to be published in the near future.

## Figures and Tables

**Table 1:** The sample codes and their designated sizes of the grinding media and ball milling durations.

Grinding Media	Sample Code	Milling Duration (min)
Various sizes (2.5 mm–11 mm)	BC	0
	30NBC	30
	60NBC	60
	90NBC	90
	150NBC	150
	180NBC	180
	360NBC	360
Uniform size (2.5 mm)	60NBC-S	60
Uniform size (11 mm)	60NBC-L	60

**Table 2:** The effect of the milling duration on the particle size of the nanobiochar. High-energy ball milling was performed using grinding media of mixed sizes.

Sample	Peaks	Size (d.nm)
BC	Peak 1	331
30NBC	Peak 1	98
60NBC	Peak 1	77
90NBC	Peak 1	35
150NBC	Peak 1	28
180NBC	Peak 1	71
360NBC	Peak 1	83
	Peak 2	687
60NBC-S	Peak 1	232
60NBC-L	Peak 1	72
	Peak 2	350

**Table 3:** The crystallite dimensions of BC and NBC at different ball milling dimensions.

Samples	2θ, ° (002)	2θ, ° (100)	FWHM (100)	Average Crystallite Diameter, L_a_ (nm)	Stacking Height, L_c_ (nm) (002)	D-Spacing, (Å) (002)
Raw	23.10	40.53	5.37	1.02	0.94	3.85
30NBC	23.29	41.12	6.94	0.84	0.93	3.82
60NBC	23.06	41.41	7.49	0.79	0.93	3.85
90NBC	23.42	41.78	7.82	0.78	0.95	3.79
150NBC	23.47	41.84	7.86	0.78	0.98	3.79
180NBC	23.77	43.74	7.28	0.83	0.98	3.74
360NBC	23.63	43.88	7.13	0.83	0.96	3.76

## Data Availability

The data presented in this study are available on request from the corresponding author.
